# Development and validation of an LC–MS/MS method for the determination of biogenic amines in wines and beers

**DOI:** 10.1007/s00706-017-1992-y

**Published:** 2017-07-11

**Authors:** Katarzyna Nalazek-Rudnicka, Andrzej Wasik

**Affiliations:** Department of Analytical Chemistry, Faculty of Chemistry, Gdańsk University of Technology, ul. G. Narutowicza 11/12, 80-233 Gdańsk, Poland

**Keywords:** Biogenic amines, Derivatization, Tosyl chloride, High performance liquid chromatography, Mass spectrometry

## Abstract

**Abstract:**

Biogenic amines are group of organic, basic, nitrogenous compounds that naturally occur in plant, microorganism, and animal organisms. Biogenic amines are mainly produced through decarboxylation of amino acids. They are formed during manufacturing of some kind of food and beverages such as cheese, wine, or beer. Histamine, cadaverine, agmatine, tyramine, putrescine, and *β*-phenylethylamine are the most common biogenic amines found in wines and beers. This group of compounds can be toxic at high concentrations; therefore, their control is very important. Analysis of biogenic amines in alcoholic drinks (beers and wines) was carried out by HPLC–MS/MS after their derivatization with *p*-toluenesulfonyl chloride (tosyl chloride). The developed method has been applied for analysis of seventeen biogenic amines in twenty-eight samples of lager beers and in twelve samples of different homemade wines (white grape, red grape, strawberry, chokeberry, black currant, plum, apple, raspberry, and quince). The developed method is sensitive and repeatable for majority of the analytes. It is versatile and can be used for the determination of biogenic amines in various alcoholic beverages.

**Graphical abstract:**

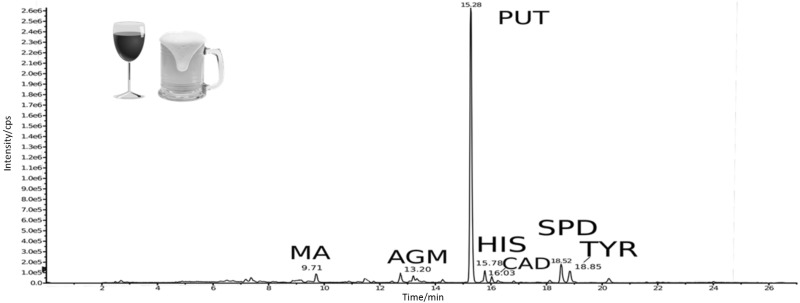

## Introduction

Biogenic amines (BA’s) are nitrogenous organic bases, occurring mainly in fermented food and beverages as a result of free amino acids bacterial decarboxylation. This reaction involves the transformation of the amino acid to biogenic amine after removal of the carboxyl group by decarboxylase enzyme or transamination of aldehydes and ketones by amino acid transaminases. In non-fermented food, biogenic amines may also occur as a result of undesirable microbial activity but at lower concentrations [1–3].

Concentrations of biogenic amines in foods (meat, cheese, beer, etc.) can be an indicator of their freshness and/or hygienic quality [4–6]. Some authors propose food quality indexes, so-called biogenic amine indexes—BAI, based on the concentrations of selected biogenic amines (e.g., cadaverine, putrescine, histamine, spermidine, spermine, and tyramine) for this purpose. In case of beers, levels of specific biogenic amines may provide an information about quality and type of the fermentation process. According to the literature, the highest content of biogenic amines is typically found in beers from spontaneous fermentation, while in case of low fermentation, biogenic amines levels are up to 20 times lower [6].

Content of biogenic amines in foods and beverages may reach levels at which they become toxic and dangerous to human health, so their determination is extremely important. Although moderate doses of biogenic amines (around 50 mg/kg food) are harmless to most people, higher amounts can cause serious consequences [6]. High content of some biogenic amines such as histamine, tryptamine, tyramine, or *β*-phenylethylamine in food and beverages may result in a range of health problems. In case of histamine poisoning, the most common symptoms among patients are nausea, diarrhea, rash, hypotension, and headache, while ‘overdose’ of tyramine may cause effects such as hypertension or migraine. It is worth to note that the presence of some of polyamines, mainly cadaverine or putrescine, may increase the toxicity of histamine and tyramine. Furthermore, polyamines such as cadaverine, putrescine, spermine, and spermidine by reaction with nitrite lead to the formation of carcinogenic nitrosamines. Additionally, the consumption of food rich in tyramine or histamine and monoamino (MAOI) or diamino (DAOI) oxidase inhibitor drugs at the same time can lead to hypertensive crisis and allergic reactions, respectively [6–10].

It is suggested that in case of alcoholic beverages, toxic impact of biogenic amines is not connected with their high concentration in these beverages, but with the consumption of large quantities of beer or wine in a very short time. Additionally, alcohol can reduce the activity of mono- and diamino oxidases (MAO, DAO), thus preventing their distribution in the organism. What is more, acetaldehyde and antidepressant drugs can also inhibit activity of these enzymes [10, 11]. Due to the above-mentioned risks, controlling the content of biogenic amines in alcoholic beverages (beers or wines) is definitely important. Additionally, biogenic amines fingerprint can be used to verify the authenticity of wines or beers and can also be used to trace their origin or in case of beers—type of fermentation [12–14].

Determination of biogenic amines in food and beverages may be problematic because of the polar nature of these compounds, low levels of concentrations and complex sample matrix [15]. To determine biogenic amines in foods, a lot of analytical techniques like gas chromatography, thin layer chromatography, capillary electrophoresis and high performance liquid chromatography had been applied. Because of high sensitivity, resolution, great versatility, and relatively simple sample preparation, HPLC seems to be one of the most extensively used techniques [1, 15]. Additionally, multiple reaction monitoring mode HPLC–MS/MS provides highly sensitive and selective detection.

Determination of biogenic amines in their native state by HPLC is difficult, because of low sensitivity and due to the severe peak tailing. To improve chromatographic behavior of these compounds, many derivatization agents have been applied [1, 15].

The choice of derivatization agent depends on detector or chromatographic technique [2]. According to the literature, the most frequently chosen derivatization agents are dansyl chloride (DNS-Cl), dabsyl chloride (DBS-Cl) benzoyl chloride, 4-chloro-3,5- dinitrobenzotrifluoride (CNBF), *o*-phthalaldehyde (OPA), diethyl ethoxymethylenemalonate (DEEMM), 6-aminoquinolyl-*N*-hydroxysuccinimidyl carbamate (AQC), 9*H*-fluoroen-9-ylmethyl chloroformate (FMOC-Cl), or tosyl chloride (TSCl). It is worth noting that the use of tosyl chloride for derivatization of monoamines is not widely encountered. There are several publications where tosyl chloride was used as derivatization agent but in most cases it was used to derivatize polyamines [9, 16–18].

The purpose of the work described in this paper was to develop an LC–MS/MS method for determination of biogenic amines in alcoholic beverages (beers and wines) after their derivatization with tosyl chloride.

## Results and discussion

To separate derivatives of fourteen biogenic amines, the gradient elution program was developed. The best results, in terms of the sensitivity and peak shape, were obtained when the mobile phase consisted of acetonitrile and water acidified with formic acid. Column temperature was set to 40 °C, in order to lower system’s backpressure.

Derivatization conditions, such as derivatization time, temperature, volumes of sample and reagents, and concentration of tosyl chloride, were optimized in terms of repeatability and efficiency (sensitivity). It was observed that in case of some compounds under the study (e.g., tryptamine and agmatine) relatively long derivatization time (120 min) was required to improve repeatability. Additionally, it was also found that stopping the derivatization reaction with 1 M HCl, as suggested Dziarkowska et al. [18], caused loss of repeatability—probably due to hydrolytic degradation of some tosyl derivatives (sulfonamides) at pH around 2.0.

Preliminary analyses of wine and beer samples were performed to select concentration ranges for six-point calibration curves. Since the content of some BA’s (isopentylamine, putrescine, tyramine, and agmatine) in beer samples was much higher than in wine samples, it was necessary to prepare two sets of calibration solutions, covering low and high concentration ranges. The calibration curves were linear within the studied concentration ranges and were characterized by coefficients of determination higher than 0.99 (Table 1).

**Table 1:** Calibration data for biogenic amines under the study

Compound	Calibration range/µg dm^−3^	Slope	Intercept	*R* ^2^	*LOD*/µg dm^−3^	*LOQ*/µg dm^−3^
Methylamine	46–830	1.613E+06	2.243E+04	0.9989	0.023	0.075
Dimethylamine	4.7–84	2.743E+04	4.158E+04	0.9983	2.9	9.6
Propylamine	110–1950	1.833E+03	5.938E+04	0.9998	24	78
Butylamine	3.6–65	1.226E+03	3.688E+03	0.9976	3.4	11
Isobutylamine	3.3–59	1.149E+03	3.976E+03	0.9998	0.82	2.8
Diethylamine	1.6–29	3.158E+03	1.506E+04	0.9997	0.44	1.4
2-Phenylethylamine	7.3–130	1.470E+03	8.361E+03	0.9978	5.4	18
Isopentylamine	3.2–58	6.278E+02	−1.137E+03	0.9982	2.7	9.0
	120–2100	5.695E+02	8.476E+02	0.9957	–	–
Tryptamine	50–910	6.546E+03	−1.934E+05	0.9998	11	36
	400–7300	4.758E+03	1.121E+06	0.9956	–	–
Cadaverine	41–730	6.594E+03	−1.685E+05	0.9996	10	34
	130–2300	4.975E+03	3.702E+05	0.9989	–	–
Putrescine	20–370	5.545E+03	4.629E+04	0.9994	7.3	24
	730–13,000	3.613E+03	8.016E+06	0.9984	–	–
Spermidine	19–340	8.481E+03	−6.489E+04	0.9999	3.6	12
	200–3600	8.298E+03	8.260E+04	0.9964	–	–
Spermine	24–440	5.914E+02	2.657E+03	0.9989	12	40
Tyramine	19–340	2.513E+03	2.019E+04	0.9979	11	36
	200–3600	1.565E+03	4.527E+05	0.9935	–	–
Histamine	9.4–170	7.327E+03	2.689E+04	0.9993	3.8	13
Hexylamine	100–1800	1.155E+03	3.688E+04	0.9995	36	120
Agmatine	100–1800	8.766E+02	1.188E+05	0.9973	83	270
	2400–44,000	5.955E+02	1.161E+06	0.9984	–	–

Repeatability of the method was estimated from triplicate analyses of wine (Table 2) and beer (Table 3) samples. For most samples, the coefficient of variation was relatively low (values were within criteria of acceptance; *CV* < 15%).

**Table 2:** Content of biogenic amines in analyzed wines (µg/dm^3^ ± standard deviation, *n* = 3)

Wine	DMA	MA	PUT	SPD	SPM	TYR
Grape_1	34.18 ± 0.80	292.2 ± 5.6	3090 ± 89	338 ± 32	N/A	232 ± 16
Grape_2	35.8 ± 2.1	282 ± 21	3333 ± 218	382 ± 18	N/A	203.9 ± 9.8
Grape (white)	N/A	170.6 ± 7.0	1697 ± 77	2592 ± 12	346.0 ± 3.5	N/A
Black currant_1	16.69 ± 0.83	264 ± 25	N/A	N/A	N/A	N/A
Black currant_2	N/A	N/A	N/A	N/A	N/A	N/A
Plum_1	13.07 ± 0.21	231 ± 19	1125 ± 15	N/A	N/A	61.0 ± 3.4
Plum_2	N/A	128.1 ± 6.4	N/A	N/A	N/A	N/A
Chokeberry	N/A	N/A	N/A	N/A	N/A	N/A
Quince	N/A	N/A	493.5 ± 6.9	73.7 ± 6.4	N/A	32.4 ± 1.3
Raspberry	28.03 ± 0.27	113.6 ± 6.1	71.9 ± 9.5	610 ± 46	N/A	N/A
Apple	69.8954 ± 0.0015	232.1 ± 4.9	N/A	106.4 ± 8.7	N/A	N/A
Strawberry	11.63 ± 0.31	292 ± 21	N/A	39.8 ± 1.8	N/A	N/A
CV^a^ [%]	0.0022–5.9	0.99–9.6	1.4–13.3	0.45–9.6	1.0	4.0–6.7

Wine	PHA	isoPEA	HIS	AGM	CAD
Grape_1	84.0 ± 7.4	53.7 ± 1.5	124.0 ± 7.6	1031 ± 40	104 ± 12
Grape_2	73.1 ± 2.4	53.0 ± 4.4	125.3 ± 4.7	1157 ± 16	107.1 ± 8.9
Grape (white)	16.3 ± 1.1	132.1 ± 4.3	55.9 ± 1.5	N/A	N/A
Black currant_1	N/A	35.26 ± 0.23	21.96 ± 0.90	N/A	N/A
Black currant_2	N/A	N/A	15.7 ± 1.9	N/A	N/A
Plum_1	37.3 ± 2.1	N/A	N/A	N/A	N/A
Plum_2	N/A	N/A	15.7 ± 1.7	N/A	N/A
Chokeberry	N/A	N/A	N/A	N/A	N/A
Quince	N/A	N/A	N/A	N/A	N/A
Raspberry	N/A	N/A	42.0 ± 1.0	N/A	N/A
Apple	31.2 ± 3.2	25.7 ± 2.6	45.62 ± 0.92	N/A	N/A
Strawberry	N/A	N/A	N/A	N/A	N/A
CV^a^ [%]	3.3–10.3	0.64–10.1	2.4–11.9	1.4–3.9	8.3–11.2

*N/A* not available

^a^Coefficient of variation CV = RSD/X_śr_ [%]

**Table 3:** Content of biogenic amines in analyzed beers (µg/dm^3^ ± standard deviation, *n* = 3)

Beer	MA	BUA	isoBUA	isoPEA	PUT	SPD
Pale lager A	78 ± 11	11.3 ± 1.6	5.76 ± 0.80	N/A	5325 ± 241	192.7 ± 5.6
Premium lager A	128 ± 14	N/A	8.62 ± 0.77	792.9 ± 7.1	7158 ± 653	739 ± 81
Strong pale lager A1	105.4 ± 6.8	N/A	7.96 ± 0.35	284 ± 11	6592 ± 126	367.2 ± 5.4
Strong pale lager A2	158.4 ± 8.9	N/A	9.36 ± 0.84	766 ± 25	8000 ± 109	781 ± 51
Standard american lager B	168.6 ± 6.5	9.5 ± 1.1	11.3 ± 1.1	1043 ± 30	7858 ± 101	218 ± 29
Strong pale lager C	107.7 ± 6.0	8.616 ± 0.067	10.362 ± 0.067	216.9 ± 6.5	6425 ± 25	381 ± 56
Standard american lager C	84.6 ± 9.2	N/A	6.61 ± 0.35	N/A	4875 ± 222	271.8 ± 8.3
Pale lager C1	N/A	N/A	7.88 ± 0.42	N/A	5908 ± 184	245 ± 20
Pale lager C2	85.7 ± 6.4	N/A	5.70 ± 0.42	N/A	5667 ± 88	260 ± 18
Pale lager D	87.4 ± 4.0	N/A	6.73 ± 0.55	293.9 ± 4.4	4567 ± 138	105 ± 11
Strong pale lager D	N/A	N/A	8.5 ± 1.0	N/A	4858 ± 250	N/A
Pale lager E	84.7 ± 2.4	N.A	7.95 ± 0.54	203.4 ± 5.3	5958 ± 142	444 ± 16
Strong pale lager F	170.4 ± 2.0	13.52 ± 0.80	16.4 ± 2.0	172 ± 10	6475 ± 87	428 ± 16
Premium lager F	198 ± 15	8.37 ± 0.67	10.02 ± 0.83	435 ± 19	6608 ± 270	638.5 ± 6.3
Pale lager F1	159.6 ± 9.7	N/A	11.31 ± 0.12	756 ± 66	7167 ± 76	721 ± 82
Pale lager F2	225 ± 11	11.7 ± 1.3	13.5 ± 1.3	560 ± 46	7300 ± 307	838 ± 59
Premium american lager G	N/A	N/A	4.429 ± 0.094	N/A	4725 ± 413	910 ± 90
Pale lager G1	N/A	N/A	N/A	N/A	3917 ± 227	653 ± 58
Pale lager G2	112.4 ± 9.3	N/A	5.55 ± 0.12	N/A	6100 ± 132	1408 ± 76
Premium lager H	124 ± 21	N/A	9.36 ± 0.90	N/A	6750 ± 71	489 ± 22
Strong pale lager I	357 ± 19	N/A	N/A	N/A	4550 ± 205	N/A
Pale lager I	193 ± 16	N/A	N/A	N/A	3700 ± 427	N/A
Non alcoholic lager J	N/A	N/A	N/A	N/A	3588 ± 371	739 ± 74
Witbier J	92.9 ± 5.8	14.09 ± 0.79	4.262 ± 0.047	742.9 ± 7.1	5958 ± 29	222.2 ± 2.5
Pale lager J1	168 ± 13	N/A	N/A	925 ± 10	7417 ± 146	1301 ± 106
Pale lager J2	108 ± 11	N/A	5.846 ± 0.071	179.9 ± 7.1	6867 ± 535	400.37 ± 0.88
Pale lager J3	N/A	N/A	5.17 ± 0.55	463 ± 20	4542 ± 138	483 ± 22
Pale lager K	186 ± 12	11.9 ± 1.1	13.7 ± 1.1	466 ± 38	8242 ± 406	1089 ± 82
CV/%	1.2–14.5	0.77–14	0.64–13.9	0.89–8.7	0.39–11.5	0.22–14.6

Beer	SPM	TYR	TRP	HIS	AGM	CAD
Pale lager A	178 ± 19	1240 ± 74	157.7 ± 5.9	N/A	N/A	238 ± 21
Premium lager A	N/A	905 ± 61	216 ± 30	66.1 ± 4.1	7257 ± 498	494 ± 27
Strong pale lager A1	187 ± 16	816 ± 93	213 ± 11	54.5 ± 2.1	5983 ± 354	303 ± 20
Strong pale lager A2	181.0 ± 9.6	1274 ± 164	235.2 ± 6.4	82.3 ± 2.8	7651 ± 209	665 ± 39
Standard american lager B	N/A	1413 ± 195	4239 ± 151	83.9 ± 4.9	11,509 ± 509	1089 ± 47
Strong pale lager C	239 ± 12	757 ± 58	195.7 ± 6.5	70.2 ± 9.1	N/A	316 ± 36
Standard american lager C	N/A	N/A	151.2 ± 9.8	N/A	N/A	428 ± 26
Pale lager C1	218.5 ± 7.9	791 ± 60	191 ± 11	66.9 ± 6.9	N/A	390 ± 18
Pale lager C2	214 ± 13	N/A	176.76 ± 0.95	N/A	N/A	185.2 ± 8.1
Pale lager D	N/A	N/A	149.4 ± 8.8	N/A	6659 ± 473	174.5 ± 5.3
Strong pale lager D	N/A	N/A	173 ± 10	N/A	N/A	210 ± 24
Pale lager E	N/A	N/A	163.0 ± 5.4	N/A	5831 ± 186	285 ± 27
Strong pale lager F	202 ± 15	N/A	191.3 ± 9.9	N/A	11,274 ± 635	291 ± 14
Premium lager F	194.7 ± 7.1	N/A	157 ± 11	N/A	10,111 ± 552	341 ± 10
Pale lager F1	218.3 ± 3.2	N/A	161.3 ± 8.5	N/A	6729 ± 433	413 ± 33
Pale lager F2	233.8 ± 4.4	N/A	188 ± 11	N/A	8385 ± 283	361 ± 18
Premium american lager G	210 ± 17	N/A	137 ± 12	N/A	5496 ± 199	N/A
Pale lager G1	152 ± 10	N/A	152.1 ± 3.8	N/A	5543 ± 634	177 ± 16
Pale lager G2	163 ± 14	N/A	171 ± 13	N/A	11,332 ± 443	219 ± 15
Premium lager H	N/A	N/A	206 ± 14	N/A	9503 ± 311	372.5 ± 2.8
Strong pale lager I	N/A	N/A	228 ± 23	N/A	N/A	463 ± 43
Pale lager I	N/A	N/A	129.1 ± 8.3	N/A	N/A	203.2 ± 1.2
Non alcoholic lager J	N/A	1720 ± 85	102.6 ± 8.4	N/A	N/A	N/A
Witbier J	N/A	12,773 ± 214	140.4 ± 2.9	93.3 ± 2.6	N/A	553 ± 29
Pale lager J1	239 ± 14	16,800 ± 140	253 ± 11	50.86 ± 0.18	11,009 ± 374	348.5 ± 5.7
Pale lager J2	178 ± 13	897 ± 44	176 ± 12	N/A	7892 ± 975	234 ± 20
Pale lager J3	N/A	12,707 ± 133	134 ± 12	N/A	N/A	181.5 ± 1.4
Pale lager K	213 ± 14	1405 ± 182	319 ± 10	48.6 ± 5.4	9847 ± 643	479 ± 47
CV/%	1.5–10.4	0.83–13.8	0.54–13.5	0.35–13	2.7–12.3	0.57–11.5

*N/A* not available

*C* coefficient of variation CV = RSD/X_śr_ [%]

Twenty-eight different commercially available beer and twelve different homemade wine samples were analyzed using the developed method. Out of seventeen biogenic amines being studied, fourteen were detected. Hexylamine, diethylamine, and propylamine were not detected in any of the analyzed wine nor beer samples.

### Homemade wines

**Fig. 1:**
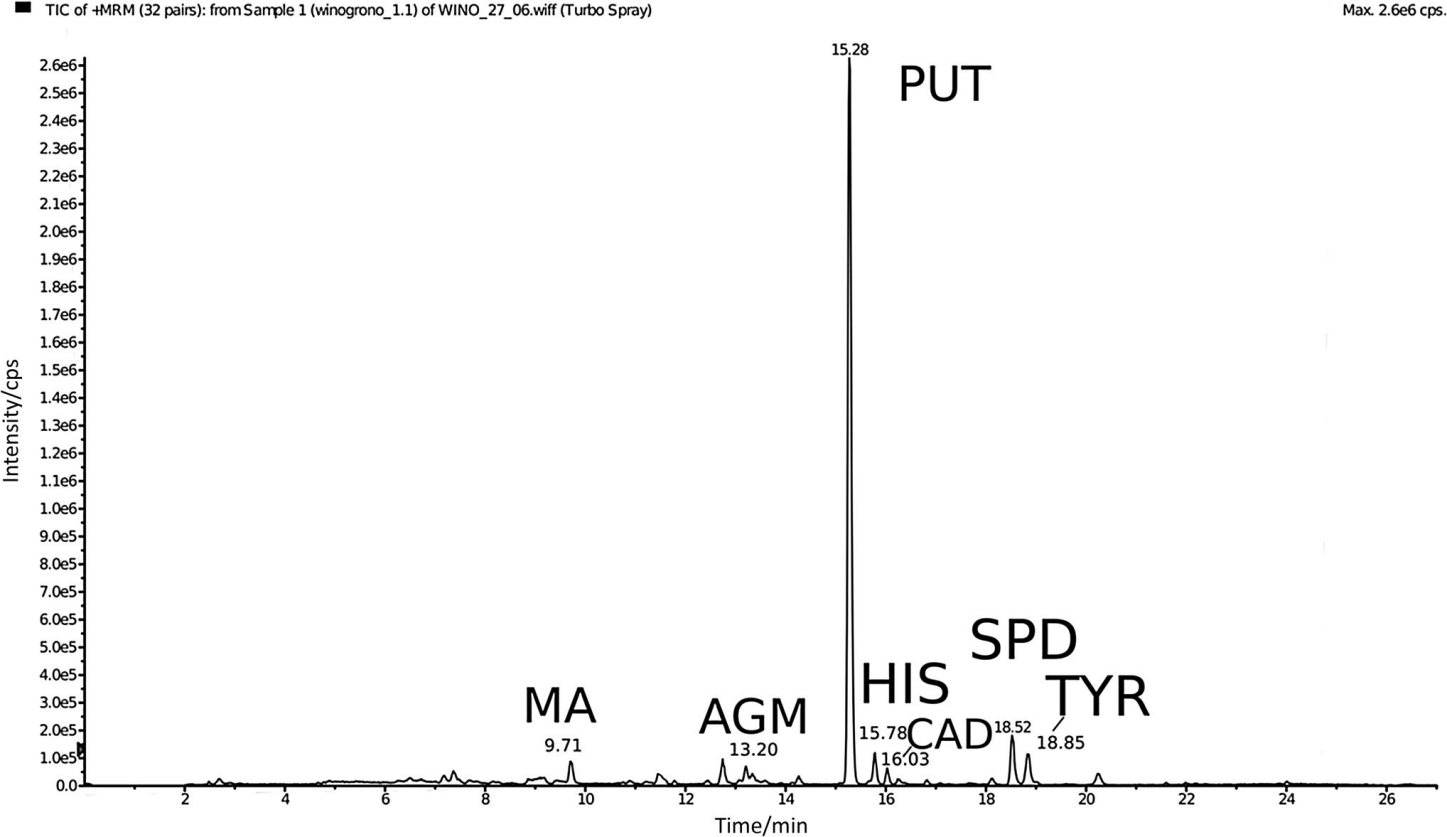
Example of total ion chromatogram obtained after analysis of red grape wine. *MA* methylamine, *AGM* agmatine, *PUT* putrescine, *HIS* histamine, *CAD* cadaverine, *SPD* spermidine, *TYR* tyramine

According to the literature, the most common biogenic amines in grape wines are tyramine, putrescine, cadaverine, 2-phenylethylamine, and histamine [19]. Analyses of our wine samples (Table 2) revealed that of all studied biogenic amines putrescine achieved the highest concentration levels (up to 3300 µg/dm^3^) followed by spermidine (up to 2600 µg/dm^3^) and agmatine (up to 1160 µg/dm^3^). Eight other amines were also detected; however, at much lower (<350 µg/dm^3^) concentration levels. These included methylamine, dimethylamine, spermine, tyramine, *β*-phenylethylamine, isopentylamine, histamine, and cadaverine. Histamine which may have adverse health effects was detected in majority of samples but at a rather low concentration levels (up to 125 µg/dm^3^ in red grape wine). The highest amounts of histamine were found in two red grape wines followed by white grape wine, apple, and raspberry wines. A typical chromatogram obtained after derivatisation and HPLC–MS/MS analysis of a wine sample is shown in Fig. 1.

The high content of putrescine in itself is not harmful, but may increase the toxicity of accompanying histamine or tyramine. Such effect can be anticipated in case of grape wines which contain the highest amounts of biogenic amines among the tested samples.

**Fig. 2:**
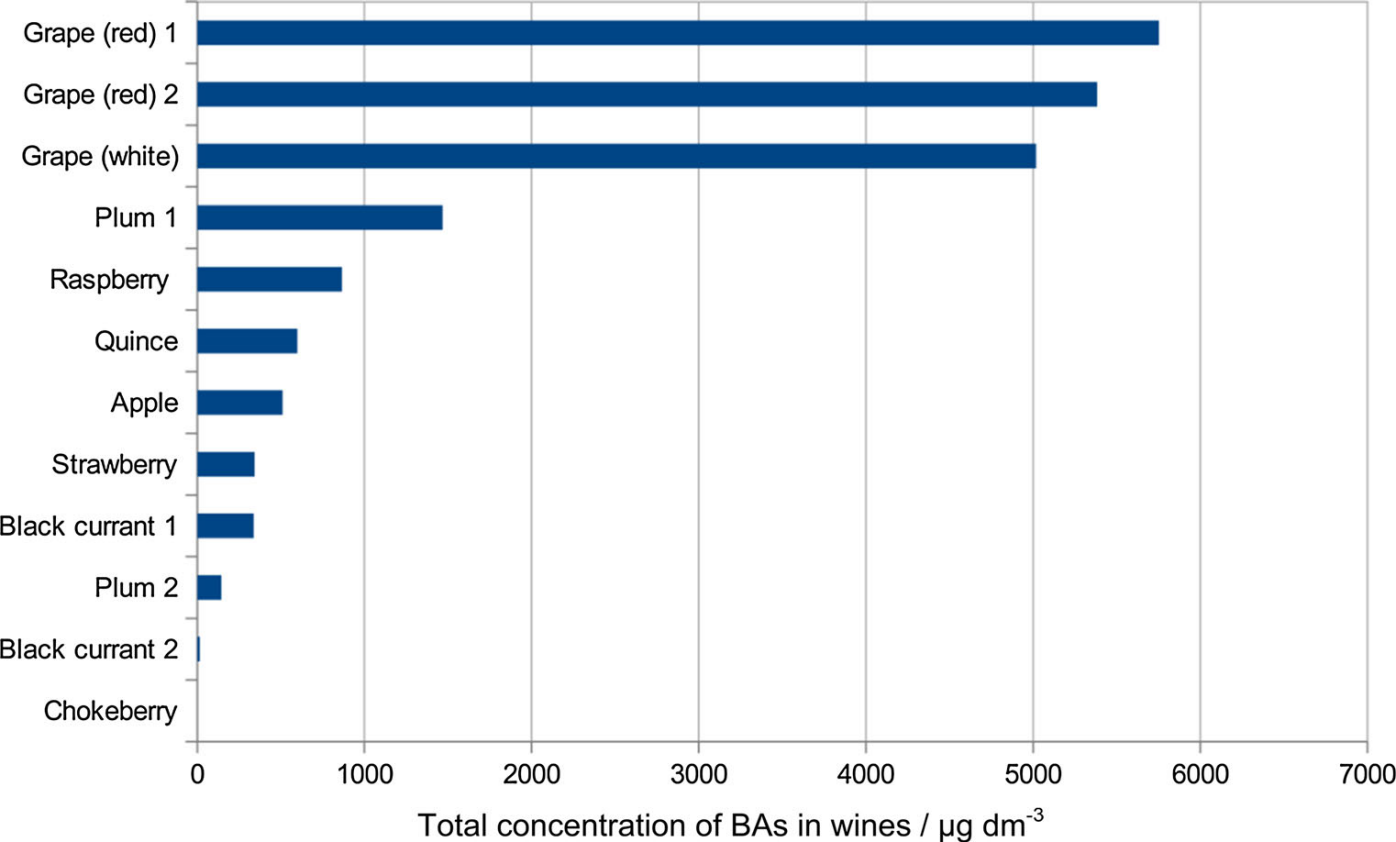
Total concentration of biogenic amines in wines/µg/dm^3^

Agmatine and cadaverine were detected only in red grape wine samples. In general, grape wines contain significantly higher amounts of biogenic amines than wines produced from other fruits (Fig. 2).

### Beer

The survey of the literature data reveals that the most dominant biogenic amines in beers are agmatine and putrescine. Additionally, histamine, tyramine, and cadaverine also often found in beers, of which tyramine and cadaverine show the greatest fluctuations [20].

**Fig. 3:**
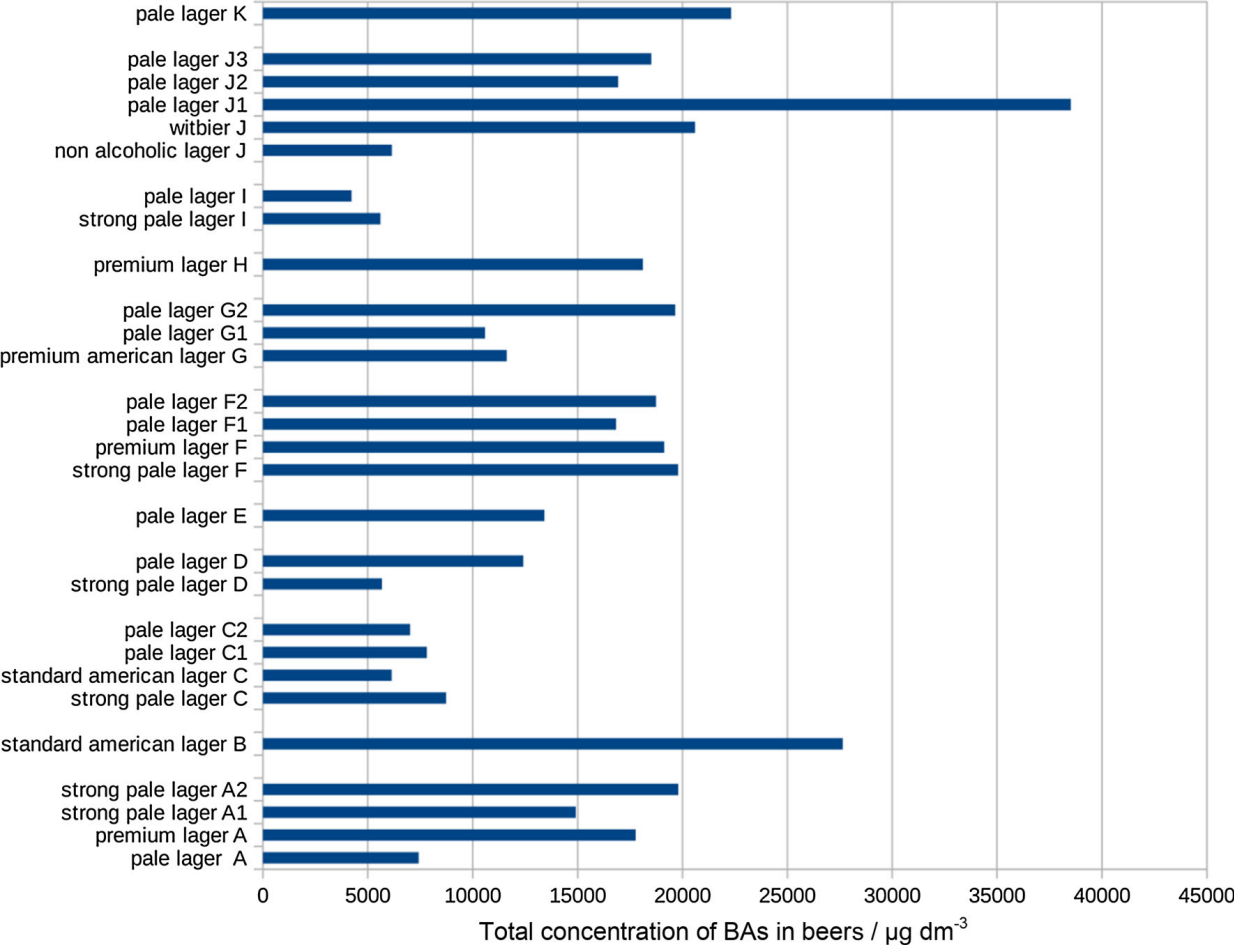
Total concentration of biogenic amines in beers/µg/dm^3^

Our study partially confirms these findings (Table 3). Total content of biogenic amines in tested samples (Fig. 3) varied from 4225 to 38,510 µg/dm^3^ (median 15,875 µg/dm^3^). The dominant biogenic amines in analyzed beers, in terms of content, were: agmatine (median 7890 µg/dm^3^), putrescine (median 6030 µg/dm^3^), and tyramine (median 755 µg/dm^3^). However, putrescine was found in all analyzed samples while agmatine and tyramine only in some of them (in 61 and 89% of samples, respectively).

Other frequently found amines were: tryptamine (100% of samples, median 170 µg/dm^3^), cadaverine (93% of samples, median 330 µg/dm^3^), and spermidine (89% of samples, median 485 µg/dm^3^). Histamine was found only in 32% of samples at relatively low concentration levels (median 65 µg/dm^3^).

An interesting observation has been made during the data analysis. Two samples of the same brand name beer sold in identical containers (Table 3, samples pale lager D and pale lager J3) drastically differed in the content of tyramine. Sample pale lager J3 contained almost 13,000 µg/dm^3^ of tyramine while supposedly the same beer (sample pale lager D), taken from another can, contained only around 375 µg/dm^3^ of this amine. Closer inspection of the two suspected containers revealed that these beers were produced in different places. It seems that the production plant may leave some kind of “amine signature” which could be used to trace the origin of the product. Verification of this hypothesis calls for more in-depth chemometric data analysis, however.

## Conclusion

A convenient method for the determination of seventeen biogenic amines in beers and wines was developed. Use of tandem mass spectrometric detection resulted in high sensitivity (LOQs at µg/dm^3^ level) and selectivity. The method is characterized by satisfactory accuracy (75–120%) and repeatability (*CV* < 15%). Derivatization procedure relies on the readily available tosyl chloride and does not require any specialized equipment. The method was successfully used for the determination of biogenic amines in the number of homemade wine and commercially available beer samples.

In general, grape wines contain around three times lower amounts of biogenic amines than beers. The amount of these amines in wines made from other fruits is even lower and strongly dependent on the type of fruit. Such information may be of a great value for those under treatment with monoamine oxidase inhibitors. Diverse levels of biogenic amines found in wines and beers may serve as their origin and originality markers. Such meta-analysis can be performed employing a chemometric approach.

## Experimental

**Fig. 4:**
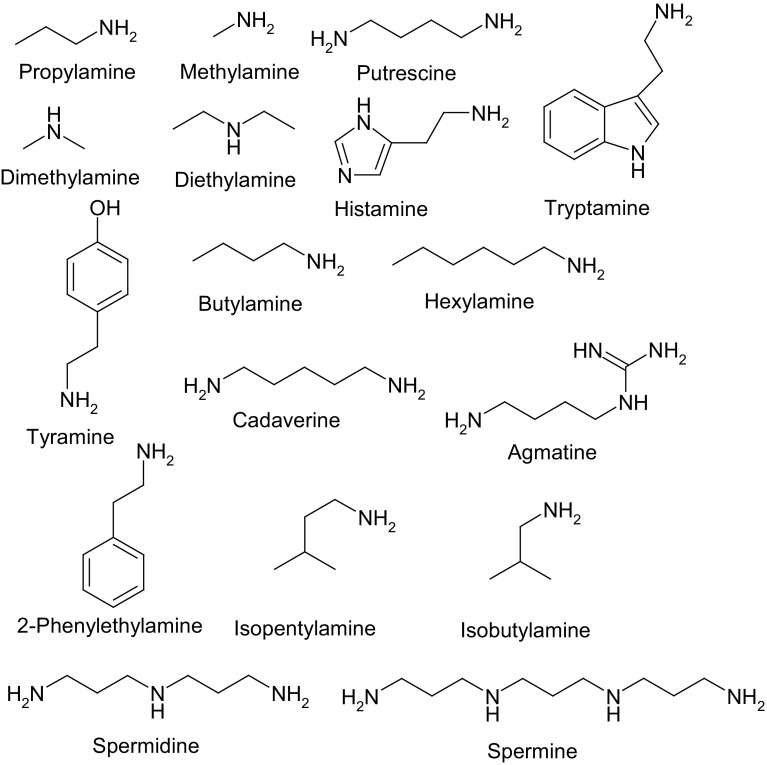
Chemical structures of biogenic amines under the study

Seventeen biogenic amines: propylamine, dimethylamine, diethylamine, methylamine, tryptamine, cadaverine, spermine, 2-phenylethylamine, tyramine, putrescine, histamine, butylamine, hexylamine, isopentylamine, isobutylamine, spermidine, agmatine, acetonitrile (LC–MS grade), and tosyl chloride (≥99%) were obtained from Sigma Aldrich (St. Louis, USA). Formic acid (FA) was purchased from Merck (Darmstadt, Germany). Boric acid and sodium hydroxide were purchased from POCH (Gliwice, Poland). Nylon Captiva Econofilters (25 mm diameter, 0.2 µm pore size) were purchased from Agilent Technologies (Santa Clara, USA). Ultrapure water was prepared using HLP_5_ system from Hydrolab (Wiślina, Poland). Borate buffer was prepared by titrating 0.5 M boric acid solution with sodium hydroxide to the required pH value. Chemical structures of studied biogenic amines are shown in Fig. 4.

### Instrumentation

The HPLC–MS/MS analyses were performed using an Agilent 1200 LC system equipped with binary pump, an online degasser, an autosampler and a thermostated column compartment equipped with a switching valve coupled with an AB Sciex 4000 QTRAP mass spectrometer. Gemini C-18 (150 × 4.6 mm, 3 µm, Phenomenex) column was used for RP-HPLC separation of the derivatives of biogenic amines. The mobile phase consisted of water containing 0.1% formic acid (component A) and acetonitrile containing 0.1% formic acid (component B). The gradient elution was as follows: 20% of B for 2 min, then linear increase to 65% B during 15 min, 65% B maintained for 3 min, increase from 65% B to 95% B during 3 min followed by 95% B maintained for 3 min. The last step was conditioning of the column for 3 min with 20% B. During the first 2 min of a run, the eluate was directed to waste by means of switching valve. The mobile phase flow rate was 1 cm^3^/min and injection volume in this case was 10 mm^3^. The column temperature throughout the separation process was kept at 40 °C. The ESI source was operated in positive ion mode with the following conditions: the source temperature was set at 500 °C, ion spray voltage was set at 5000 V, nebulizer gas was set at 45 psi, and heater gas and curtain gas were set 30 and 20 psi, respectively. Source and MS parameters are shown in Table 4. To acquire chromatograms and control instrumentation, Analyst Software version 1.5.2 (AB Sciex, CA, USA) was used.

**Table 4:** MRM transition parameters for detection of amine’s tosyl derivatives

Compound	MRM^a^	Declustering potential/V	Collision energy/V
Methylamine	186.101 → 155	71	17
	186.101 → 91	71	29
Dimethylamine	200.121 → 155	76	25
	200.121 → 91	76	37
Propylamine	214.095 → 155.10	71	23
	214.095 → 91	71	37
Butylamine	228.144 → 155	66	23
	228.144 → 91	61	37
Isobutylamine	228.144 → 155	66	23
	228.144 → 91	61	37
Diethylamine	228.144 → 155	66	23
	228.144 → 91	61	37
2-Phenylethylamine	276.127 → 105	66	23
	276.127 → 77	66	69
Isopentylamine	242.157 → 155	66	27
	242.157 → 91	66	43
Tryptamine	315.060 → 144.10	51	17
	315.060 → 117	51	77
Cadaverine	411.10 → 240.20	96	23
	411.10 → 184.10	96	29
Putrescine	397.11 → 226.30	96	23
	397.11 → 155.10	96	35
Spermidine	608.284 → 383.20	91	31
	608.284 → 226.30	106	37
Spermine	819.336 → 212.100	126	49
	819.336 → 281.100	126	51
Tyramine	446.172 → 275.100	101	21
	446.172 → 155.000	101	35
Histamine	420.095 → 109.000	81	35
	420.095 → 91.000	81	67
Hexylamine	256.190 → 155.100	71	27
	256.190 → 91.000	71	41
Agmatine	439.218 → 155.200	121	37
	439.218 → 91.000	121	71

Condition of ESI source: Source temperature 500 °C, nebulizer gas 45 psi, heater gas 30 psi, curtain gas 20 psi, capillary voltage 5000 V

### Beer and wine sample collection and preparation

Twenty-eight of sample beers (type of lager) differing in place of production and the alcohol content and eighteen samples of wine differing in the main material (grape, black currant, plum, apple, chokeberry, quince, raspberry, white grape, strawberry) were purchased at local supermarkets.

All beers and wines were analyzed within one day from purchase. In case of beer, samples were degassed in an ultrasonic bath for 10 min and diluted (1 + 4, v/v) with ultrapure water, while in case of wine, degassing step was omitted and the wine samples were diluted (1 + 9, v/v) with ultrapure water.

**Fig. 5:**
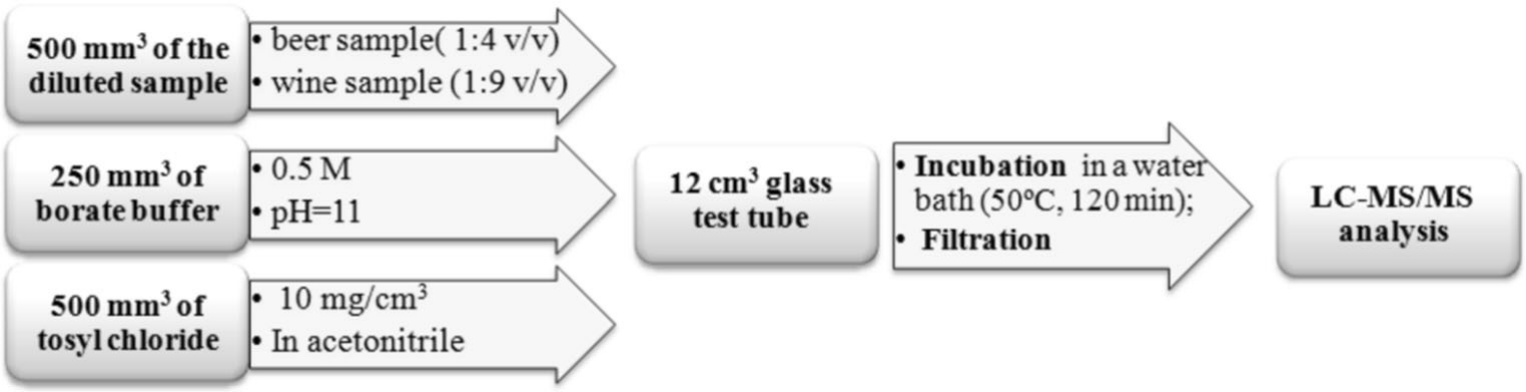
Diagram illustrating derivatization of wine and beer samples

To perform derivatization, 500 mm^3^ of diluted beer or wine sample was transferred to a 12 cm^3^ glass test tube and mixed with 250 mm^3^ of borate buffer (0.5 M, pH = 11) and 500 mm^3^ of tosyl chloride solution (10 mg/cm^3^, in acetonitrile). After mixing, samples were incubated for 120 min at 50 °C in water bath. Finally, the samples were filtered through a 0.2 µm nylon filter (Agilent Technologies, Santa Clara, CA, USA) and injected into chromatographic system. Overall way for the preparation of beer and wine samples is shown in Fig. 5.

### Calibration curves

Stock solutions (1 mg/cm^3^) of each biogenic amine were prepared in 0.1 M HCl. Then, in order to produce the standard mix, relevant aliquots of each of seventeen solutions were introduced into the 25 cm^3^ volumetric flask and made up to the mark with acetonitrile: 0.1 M HCl (3 + 7, v/v) mixture. The concentrations of each biogenic amine have been adjusted on the basis of preliminary analyses of the samples. Standard mix prepared in this way has been used to prepare the calibration curves. Six points (each point in triplicate) calibration curves were prepared by diluting variable aliquots of the standard mix with acetonitrile: 0.1 M HCl (3 + 7, v/v) mixture. Thereafter, 100 mm^3^ of each calibration solution was mixed with 400 mm^3^ of ultrapure water and subjected to the same derivatization procedure as beer and wine samples.

**Fig. 6:**
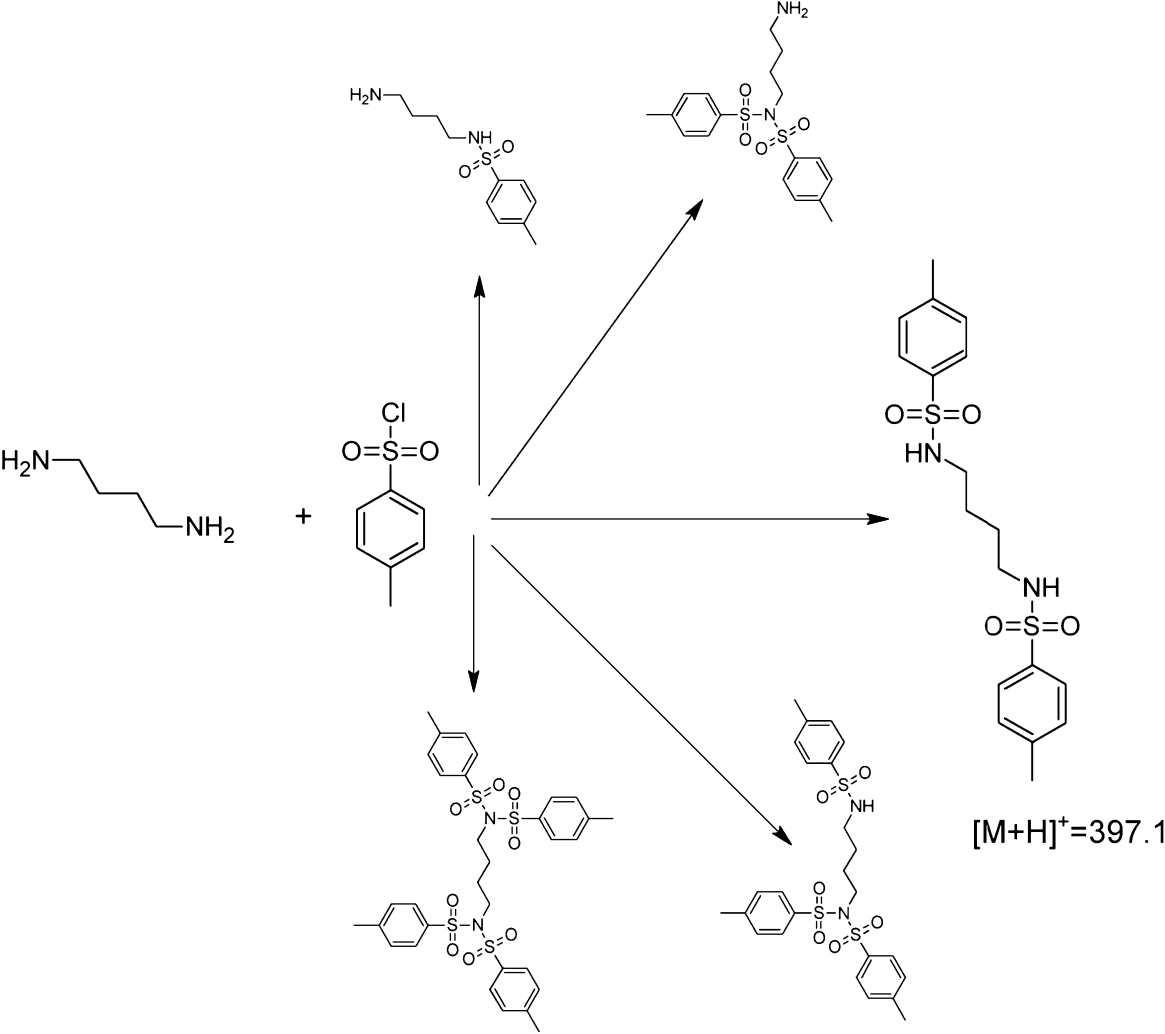
Formation of putrescine’s tosyl derivatives (tentative, favored structure enlarged)

One must be aware that the derivatization reaction may lead to the formation of different, multiply tosylated amine derivatives. Formation of these derivatives is influenced by different factors, steric effect being probably the most important one. During the development of the method described here, every single amine has been subjected to the derivatization and the product giving the most intense spectrometric peak has been selected. It turned out that substitution of single hydrogen, bound to the primary (or secondary) nitrogen atom proceeds fastest in most cases. The derivatization scheme (with putrescine as an example) is presented in Fig. 6.

### Matrix effects

**Fig. 7:**
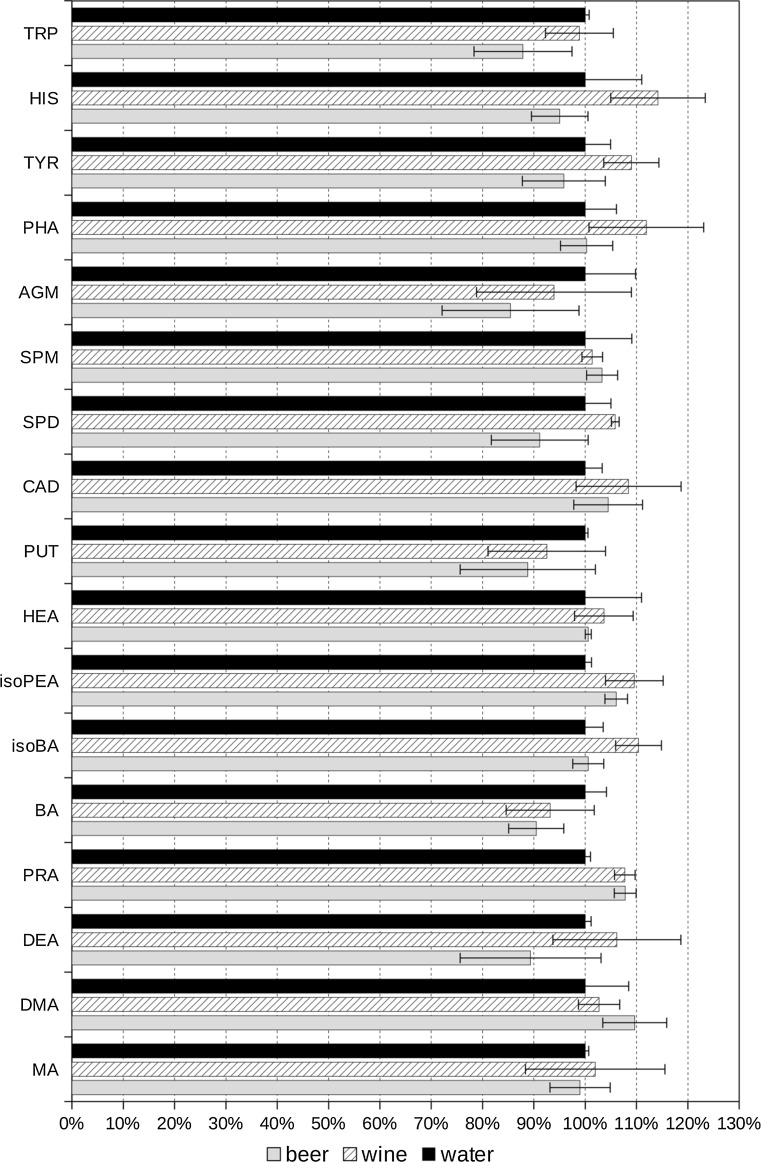
Estimation of matrix effects. *Slopes* of regression *lines* calculated after spiking the samples of wine and beer with mixture of BA’s standards were normalized against the water sample

The impact of sample matrix on derivatization yield was estimated using standard addition method. Three kinds of samples were derivatized: water, wine, and beer. The samples (each in triplicate) were spiked at three different concentration levels with the same amount of standard mixture of biogenic amines. The average peak area corresponding to each analyte was plotted against the added amount of the analyte. Linear regression lines were calculated and their slopes compared. The results of this comparison are shown in Fig. 7. In general, it seems that wine matrix slightly increases derivatisation yield while beer matrix acts just opposite. The observed effects are more or less randomly visible among different biogenic amines derivatives (characterized by different retention times); therefore, in our opinion the core cause of this enhancement or inhibition lies in the derivatization reactions. The exact mechanism is unknown at the time of writing but certainly it is worth further studies.
